# Rescue cholangioscopy using a novel ultra-flexible cholangioscope for a tortuous cystic duct stump calculus

**DOI:** 10.1055/a-2936-4171

**Published:** 2026-08-25

**Authors:** Ankoor H. Patel, Petros Benias, Michael Ma, Arvind J. Trindade

**Affiliations:** 1 Medicine, Division of Gastroenterology 12287 Rutgers University School of Medicine, RWJBarnabas HealthNew BrunswickNJUnited States

Cystic duct remnant stones, after cholecystectomy, are challenging to treat. 1​ Endoscopic retrograde cholangiopancreatography (ERCP) is the primary endoscopic approach, 2​ but standard techniques often fail due to anatomical factors such as the remnant size, tortuosity, stone location, impaction, and inflammation.

**Fig. 1:**
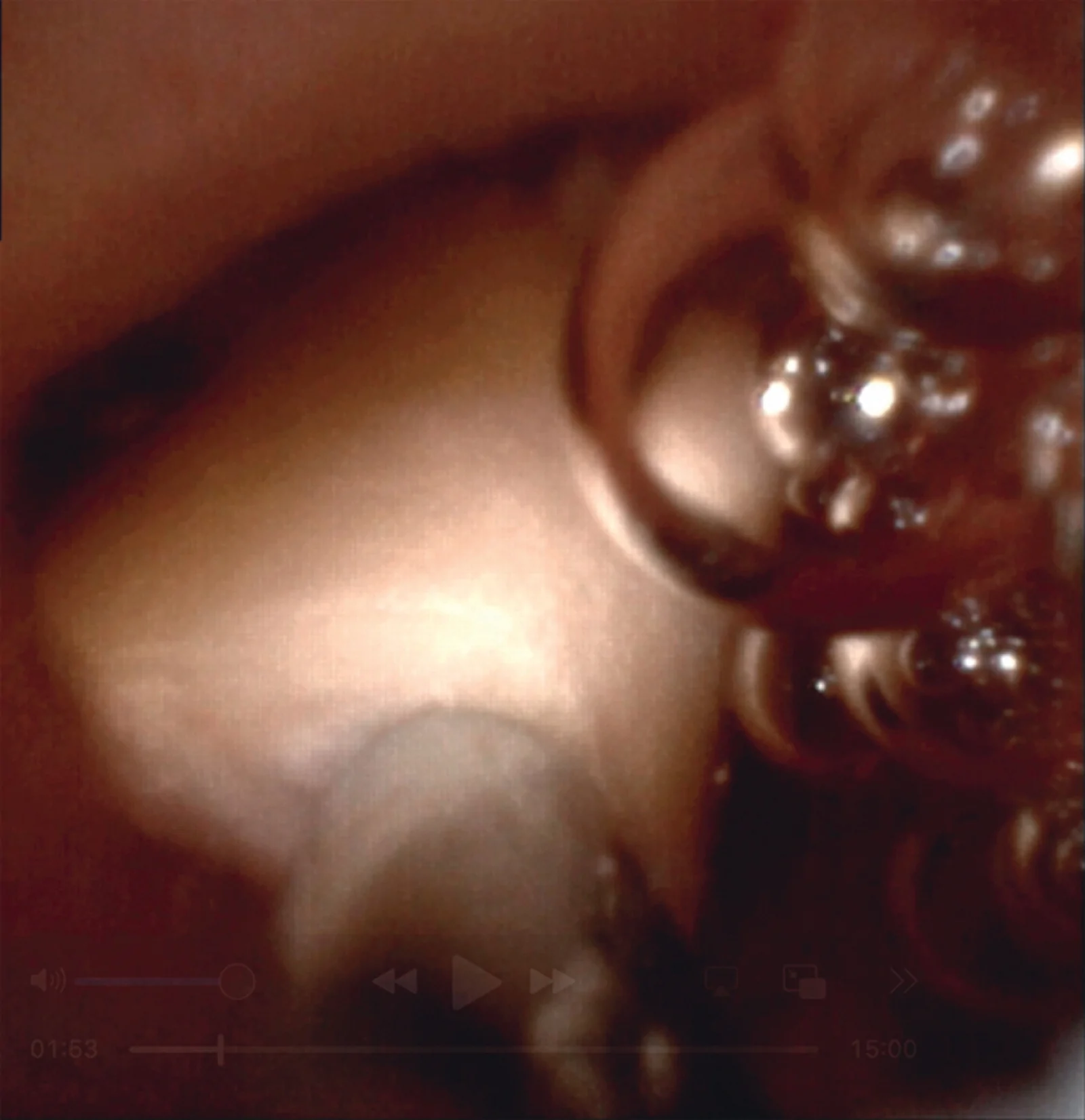
Electrohydraulic lithotripsy of a cystic duct stone performed using an ultra-flexible cholangioscope (Dragonfly Pancreaticobiliary Endoscope, Medtronic, Minneapolis, MN).

**Fig. 2:**
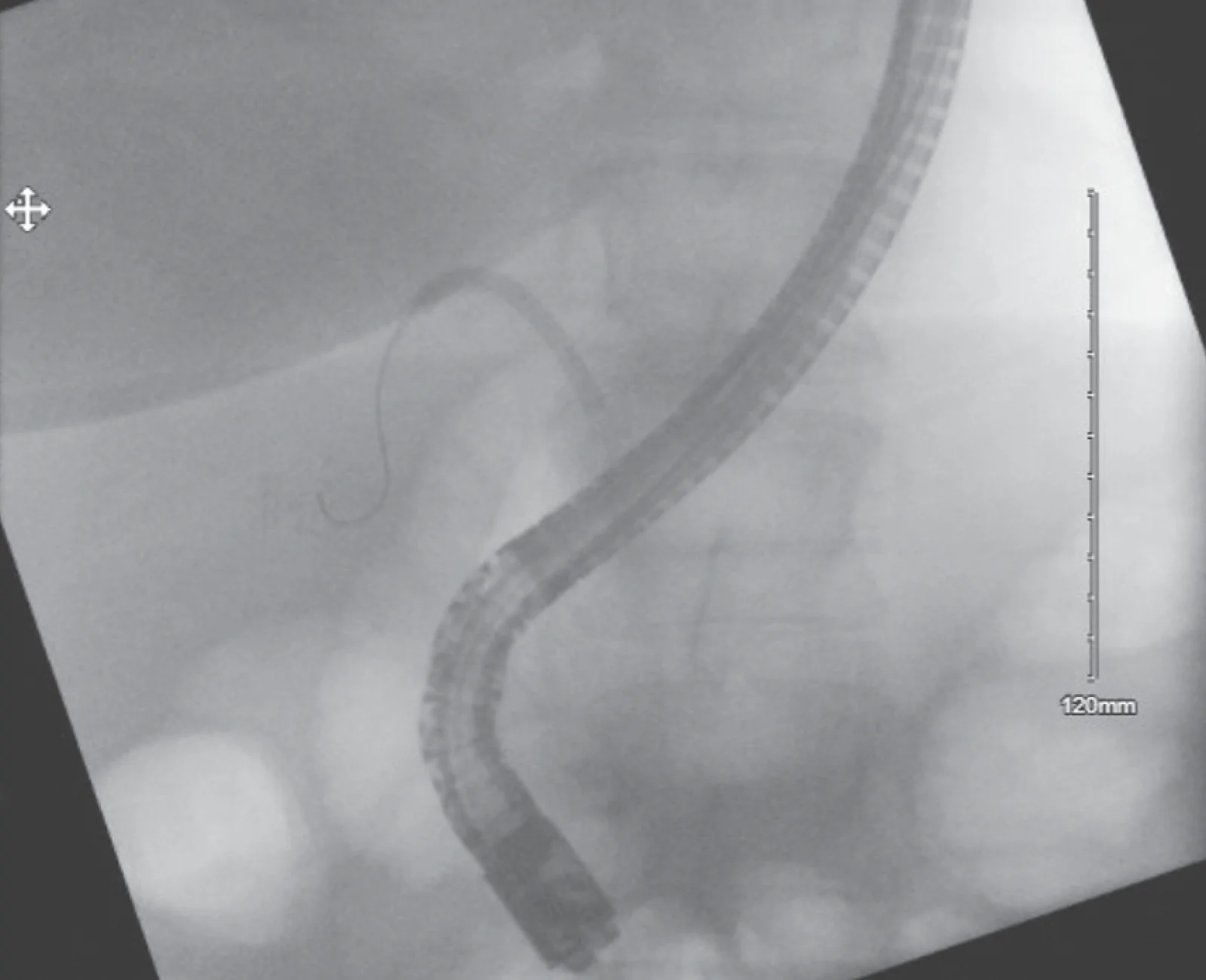
A fluoroscopic view of the cholangioscope traversing and navigating through the remnant cystic duct.

**Fig. 3:**
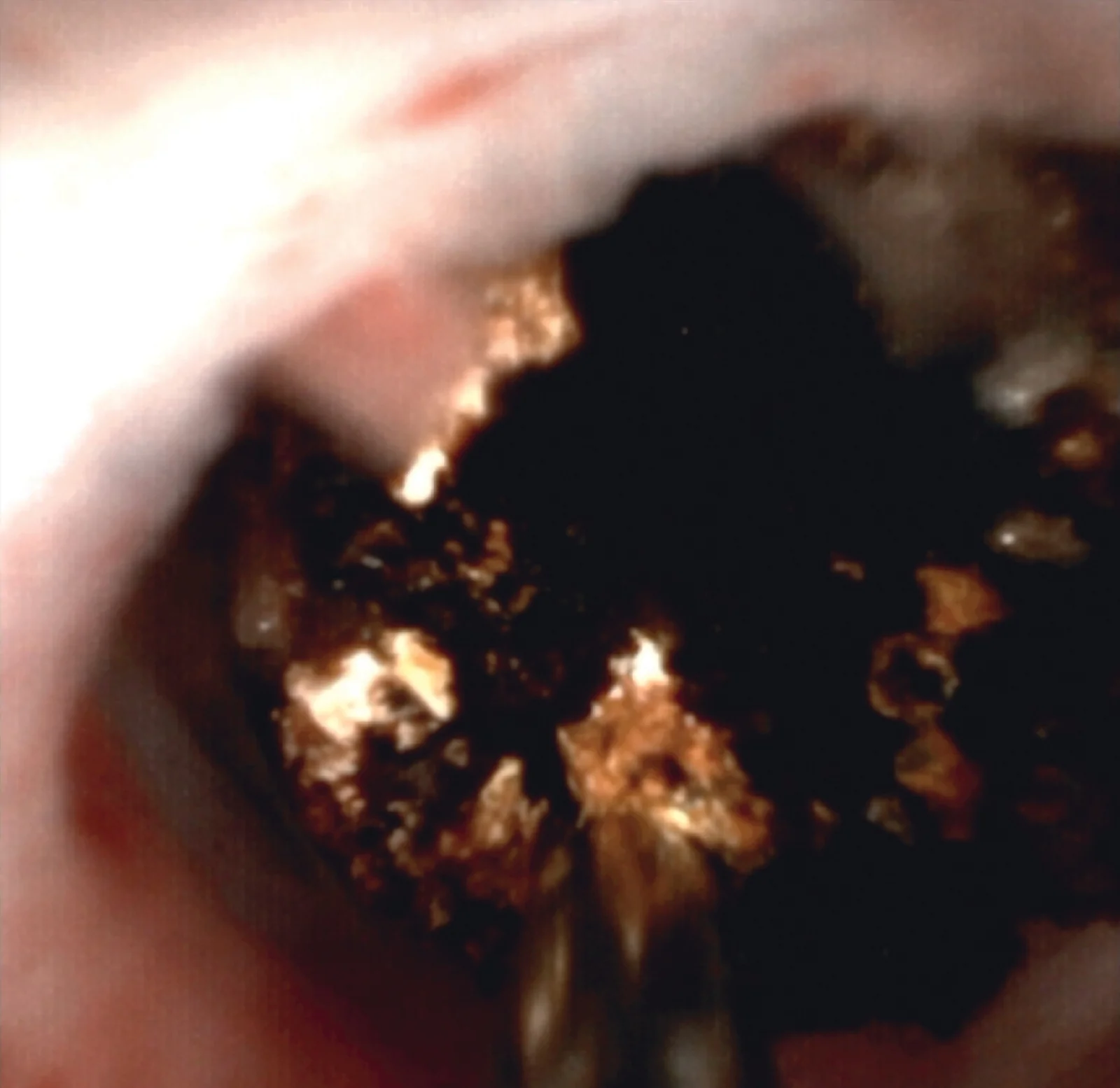
Electrohydraulic lithotripsy of an impacted cystic duct stone via a flexible cholangioscope, following the successful navigation of the tortuous duct.

We present two cases of cystic calculi successfully managed with a novel ultra-flexible cholangioscope (Dragonfly Pancreaticobiliary system, Medtronic, Minneapolis, MN). Our first case is a 41-year-old woman who presented to the emergency department with acute on-chronic abdominal pain and magnetic resonance imaging/magnetic resonance cholangiopancreatography (MRI/MRCP) revealed a cystic duct remnant with an obstructing stone and signs of stumpitis. The case was initially performed using our standard cholangioscope (Spyglass DS, Boston Scientific, Marlborough, MA). However, due to the cholangioscope’s shaft stiffness and the duct’s tortuosity, the stone could not be reached for electrohydraulic lithotripsy (EHL). In the same endoscopic session, the ultra-flexible cholangioscope was used. This scope traversed the tortuous duct ( Fig. 1 ) and reached the stone ( Fig. 2 ). Successful EHL was performed on the impacted stone, and a stent was placed due to severe inflammation of the cystic stump caused by the stone. Our second case is a 59-year-old man with post-cholecystectomy who presented with abdominal pain. MRI/MRCP showed stones within the cystic duct remnant. ERCP using the novel ultra-flexible cholangioscope was performed ( Video 1 ). EHL of two stones ( Fig. 3 ), followed by basket retrieval of a third stone was performed. A stent was placed due to stumpitis.

**Video 1** Two cases demonstrating the successful management of cystic duct remnant stones with a novel ultra-flexible cholangioscope.


These two cases highlight how the novel ultra-flexible cholangioscope can be used to access tortuous ducts such as the cystic duct. The ultra-flexible shaft, combined with the ability to rotate 360°, allows navigation through difficult anatomy. Combining these features with a 1.7 mm diameter channel allow for EHL with a large diameter probe. This represents an approximately 42% larger channel than conventional single-operator cholangioscopes (typically 1.2 mm), permitting the use of more robust EHL/laser probes and larger baskets without increasing the scope’s outer profile. Our cases suggest that this novel cholangioscope’s flexibility and larger channel may extend these benefits to cases where earlier-generation devices fail.

Endoscopy_UCTN_Code_TTT_1AR_2AL
